# Evaluating Single‐Domain and Multi‐Domain Interventions on Global Cognition in Cognitively Unimpaired Older Adults: A Network Meta‐Analysis

**DOI:** 10.1002/alz70860_106615

**Published:** 2025-12-23

**Authors:** Augusto J. Mendes, Federica Ribaldi, Ozge Sayin, Roberta Mulargia, Gabriele Volpara, Stefano Giannoni‐Luza, Giulia Remoli, Giorgi Khachvani, Umberto Nencha, Stefano F Cappa, Giovanni B. Frisoni

**Affiliations:** ^1^ Geneva Memory Center, Department of Rehabilitation and Geriatrics, Geneva University Hospitals, Geneva, Geneva, Switzerland; ^2^ Laboratory of Neuroimaging of Aging (LANVIE), University of Geneva, Geneva, Switzerland; ^3^ Università degli studi di Cagliari, Cagliari, Italy; ^4^ University Institute of Advanced Studies of Pavia, Pavia, Italy; ^5^ Center for Neurodegenerative Diseases and the Aging Brain, University of Bari ‘Aldo Moro’, Bari, Italy; ^6^ Fondazione IRCCS San Gerardi Dei Tintori, Monza, Italy; ^7^ Geneva Memory Clinic, Department of Rehabilitation and Geriatrics, Geneva University Hospitals, Geneva, Switzerland; ^8^ Institute for Advanced Studies, IUSS, Pavia, Italy

## Abstract

**Background:**

Dementia incidence has been partially attributed to modifiable vascular and lifestyle‐related risk factors, highlighting the potential for prevention through targeted interventions. Both single‐ and multidomain interventions addressing these risk factors have been proposed to mitigate cognitive decline. However, the effectiveness of these strategies in preventing dementia among cognitively unimpaired individuals remains unclear. To better understand their comparative efficacy, this study aims to evaluate and compare the effectiveness of single‐ and multidomain interventions in maintaining or improving global cognition among cognitively unimpaired older adults.

**Method:**

We conducted a systematic review and network meta‐analysis of prospective interventional studies involving cognitively unimpaired adults aged 50 and above. Studies were included if they assessed global cognition as an outcome and involved interventions targeting diet (D), physical activity (PA), cognitive training (CT), social activity (SA), or health monitoring/advice (H). Effect sizes (standardized mean differences; SMD) were calculated for each comparison based on their direct and indirect effect, and subgroup analyses were performed for long‐term interventions (>6 months).

**Result:**

A total of 120 studies encompassing 18021 participants met the inclusion criteria. Our analysis revealed that multidomain interventions combining PA and CT demonstrated the largest effect size (SMD = 0.27; 95%CI = 0.12‐0.42), followed by single‐domain CT (SMD = 0.17; 95%CI = 0.06‐0.28), PA+CT+H (SMD = 0.16; 95%CI = ‐0.12‐0.44), D+PA+CT+H (SMD = 0.14; 95%CI = 0.01‐0.27), and single‐domain PA (SMD = 0.12; 95%CI = 0.04‐0.19). Subgroup analyses of long‐term interventions showed larger effect sizes, particularly for single‐domain CT (SMD = 0.34; 95%CI = 0.10‐0.58), PA+CT (SMD = 0.26; 95%CI = ‐0.01‐53), SA (SMD = 0.26; 95%CI = ‐0.11‐0.62), and D+PA+CT+H (SMD = 0.20; 95%CI = 0.01‐0.38).

**Conclusion:**

This network meta‐analysis underscores the superior efficacy of multidomain interventions, particularly those combining PA and CT, in improving/maintaining global cognition among older adults. Long‐term interventions amplify these benefits, reinforcing the importance of sustained, comprehensive approaches in addressing cognitive health. These results provide valuable insights for designing future prevention strategies targeting modifiable vascular and lifestyle‐related risk factors.

